# *BraggNN*: fast X-ray Bragg peak analysis using deep learning

**DOI:** 10.1107/S2052252521011258

**Published:** 2021-12-10

**Authors:** Zhengchun Liu, Hemant Sharma, Jun-Sang Park, Peter Kenesei, Antonino Miceli, Jonathan Almer, Rajkumar Kettimuthu, Ian Foster

**Affiliations:** a Data Science and Learning Division, Argonne National Laboratory, Lemont, IL 60439, USA; b X-ray Science Division, Argonne National Laboratory, Lemont, IL 60439, USA

**Keywords:** materials science, high-pressure powder diffraction, WAXS, X-ray microscopy, structure prediction

## Abstract

X-ray diffraction based microscopy techniques such as high-energy diffraction microscopy (HEDM) rely on knowledge of the position of diffraction peaks with high precision. These positions are typically computed by fitting the observed intensities in detector data to a theoretical peak shape such as pseudo-Voigt. As experiments become more complex and detector technologies evolve, the computational cost of such peak-shape fitting becomes the biggest hurdle to the rapid analysis required for real-time feedback in experiments. To this end, we propose *BraggNN*, a deep-learning based method that can determine peak positions much more rapidly than conventional pseudo-Voigt peak fitting. When applied to a test dataset, peak center-of-mass positions obtained from *BraggNN* deviate less than 0.29 and 0.57 pixels for 75 and 95% of the peaks, respectively, from positions obtained using conventional pseudo-Voigt fitting (Euclidean distance). When applied to a real experimental dataset and using grain positions from near-field HEDM reconstruction as ground-truth, grain positions using *BraggNN* result in 15% smaller errors compared with those calculated using pseudo-Voigt. Recent advances in deep-learning method implementations and special-purpose model inference accelerators allow *BraggNN* to deliver enormous performance improvements relative to the conventional method, running, for example, more than 200 times faster on a consumer-class GPU card with out-of-the-box software.

## Abstract 2

We propose *BraggNN*, a deep-learning based method, to accelerate the most computation-intensive part of polycrystal diffraction data analysis (diffraction signal characterization). The application of *BraggNN* for real experimental data demonstrates that it can deliver consistent (sometimes even slightly better) results compared with the conventional method while running hundreds of times faster.

## 1. Introduction

Advanced materials affect every aspect of our daily lives, including the generation, transmission and use of energy. Accelerating the pace of materials design promises to enhance economic activity and the transition to a cleaner energy future. However, current material design approaches rely heavily on intuition based on past experiences and empirical relationships. In order to qualify new materials for critical applications, several high-energy X-ray characterization methods have been developed over the past decade. One of the foremost is high-energy diffraction microscopy (HEDM) (Park *et al.*, 2017 ▸), which provides non-destructive 3D information on a structure and its evolution within polycrystalline materials. HEDM techniques have enabled breakthroughs in understanding of various processes, through carefully designed experiments that are tractable for analysis by researchers (Naragani *et al.*, 2017 ▸; Bernier *et al.*, 2020 ▸; Wang *et al.*, 2020 ▸). These methods use diffraction and tomographic imaging of up to centimetre-sized objects with resolutions down to the micrometre level.

**Figure 1:**
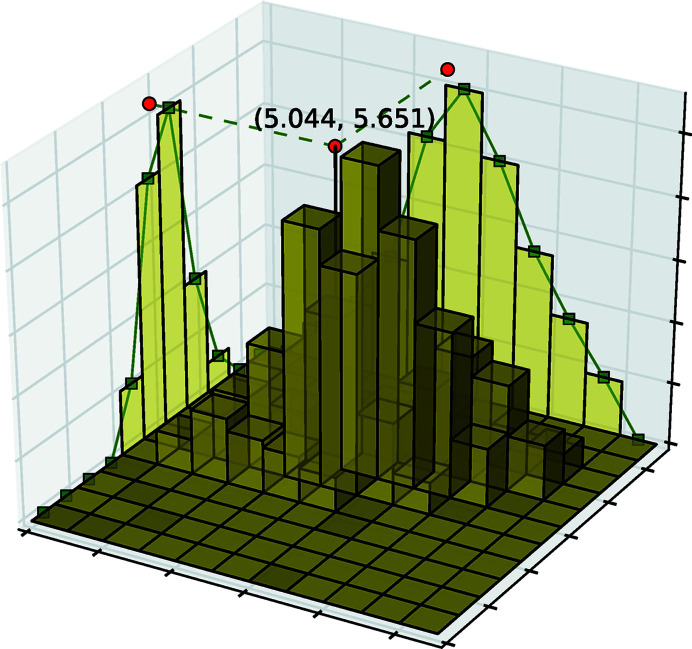
Diffraction peak in X-ray diffraction (11 × 11 patch). The height denotes photon counts, and the red dots show the peak position computed by fitting a pseudo-Voigt profile.

A conventional HEDM experiment involves four steps: (1) data acquisition, (2) transfer of full scan from detector to central storage, (3) offline Bragg peak analysis to determine precise peak positions and (4) reconstruction of grain information from the Bragg peak positions generated in the third step (Sharma *et al.*, 2012*a* ▸,*b* ▸). A single typical HEDM scan involves acquiring diffraction images (1440–3600 frames in total) while rotating the specimen at a constant speed about an axis (similar to tomography) while being illuminated by an X-ray beam perpendicular to the rotation axis. Data acquisition is increasingly fast: a single typical HEDM scan consisting of 1440–3600 frames takes about 6–15 min to acquire today at the Advanced Photon Source (APS) and projected to be 50–100 s after the planned upgraded of APS (APS-U) (Streiffer *et al.*, 2015 ▸) with faster detectors. Rotation of the specimen enables each grain to satisfy the Bragg-diffraction condition multiple times, resulting in multiple diffraction peaks from the grain. Reconstruction of far-field (FF) HEDM data depends on determination of the peak positions with sub-pixel accuracy, which can deviate significantly from the maxima as shown in Fig. 1 ▸.

Peak positions are typically computed by (optionally) transforming the peaks to polar coordinates and then fitting the peaks to a pre-selected peak shape such as Gaussian, Lorentzian, Voigt or Pseudo-Voigt (Sharma *et al.*, 2012*a* ▸). The Voigt profile, a probability distribution given by a convolution of a Cauchy–Lorentz distribution and a Gaussian distribution, is often used in analyzing data from spectroscopy or diffraction. However, it is computationally expensive to fit a Voigt profile in 2D (or 3D) space for each Bragg peak, so the peak shape is approximated to a pseudo-Voigt profile. Depending on sample properties and the extent of the mechanical, thermal, electromagnetic or chemical stimuli applied to the sample, processing time can range from 10 min to a few weeks, even when using an HPC cluster with thousands of CPU cores. These long data analysis times are more than just an inconvenience: they prevent experimental modalities that depend on measurement-based feedback for experimental steering.

Although we describe the *BraggNN* framework as applied to FF-HEDM, it is also useful for other diffraction techniques dealing with single or polycrystal diffraction. The data and source code that support the findings of this study are openly available at https://github.com/lzhengchun/BraggNN.

## 2. *BraggNN* and its training

A significant fraction of HEDM data analysis time is spent on determining peak positions, motivating us to seek methods for accelerating this operation. Artificial neural networks are known for their universal approximation capability that allows them to represent complex and abstract functions and relationships (Hornik, 1991 ▸). Thus, a promising solution to the Bragg peak localization challenge is to train a deep-learning (DL) model to directly approximate the position of Bragg peaks. Advances in both machine-learning (ML) methods and AI inference accelerators allow such a model to run much faster than conventional methods (Abeykoon *et al.*, 2019 ▸), making it feasible to extract peak information from streaming data in real-time, enabling rapid feedback and reducing downstream transfer, storage and computation costs.

### 2.1. Model design

DL is part of a broader family of ML methods based on artificial neural networks to progressively extract higher level features from the pixel-level input through a hierarchical multi-layer framework. The convolutional neural network (CNN), a widely used building block of DL models for visual image analysis, is an efficient parameter owing to the translation-invariant property of its representations, which is the key to the success of training DL models without severe over-fitting. Although a strong theory is currently missing, much empirical evidence supports the notion that both the translation-invariant property and convolutional weight sharing (whereby the same weights are shared across an entire image) are important for good predictive performance (Cheng *et al.*, 2017 ▸).

In this work, we express this task as a regression problem using supervised ML and present *BraggNN*, a deep neural network-based model for precisely localizing Bragg peaks far more rapidly than that which can be achieved by applying conventional fitting methods to peak-shape profiles. Note that we are not concerned here with the problem of locating within an image a patch that contains a peak (the ‘object localization problem’) because Bragg peaks are easily separated from background using a heuristic thresholding value, and from neighbor peaks using a connected-component labeling algorithm (overlapped peaks and fitting in 3D will be studied in future work) (Fiorio & Gustedt, 1996 ▸; Wu *et al.*, 2005 ▸). Our problem rather is to determine, with sub-pixel precision, the center-of-mass (COM) of a diffraction peak in a supplied patch: the ‘peak localization problem’.

**Figure 2:**
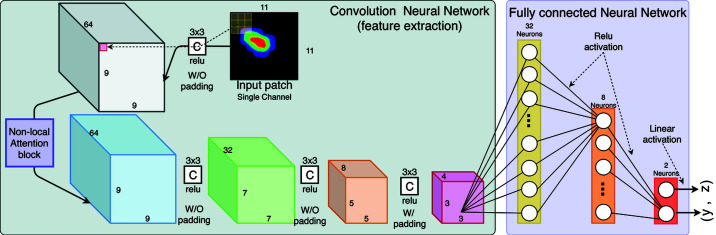
Application of the *BraggNN* deep neural network to an input patch yields a peak center position (*y*, *z*). All convolutions are 2D of size 3 × 3, with a rectifier activation function. Each fully connected layer, except for the output layer, also has a rectifier activation function. The non-local attention block will be discussed in Appendix A[App appa].

The *BraggNN* network architecture, shown in Fig. 2 ▸, comprises a series of CNN layers (four in the figure) acting as feature extractors, followed by a series of fully connected layers (three in the figure) that generate a regression prediction. Each CNN kernel/filter is an artificial neuron that, in contrast to traditional algorithms in which kernels are hand-engineered, learns to extract a type of feature (*e.g.* various oriented edges, or blobs of color) from its input. Each 2D CNN neuron has 3 × 3 × *c* learnable weights plus one learnable bias to convolve a feature map (a 3D volume shaped as height × width × depth/channel) with *c* channels (*e.g.* the input patch has one channel as shown in the figure).

Here we use the first layer, which takes a Bragg peak in a patch with 11 × 11 × 1 (*c* = 1) pixels as input and outputs 64 feature maps (each has 9 × 9 pixels) as an example to explain the convolution operation. At every convolution position, for example the one shown as a dotted line in the figure, the dot product between the weights and the input entry (3 × 3 × *c* centered at the convolution position) is computed and added to the learnable bias. This convolution result, called the activation, is then passed through a rectified linear unit [ReLU, *f*_relux_ = max(*x*, 0)] activation function to yield a feature. Each kernel is convolved (vertically and horizontally) across the input image, producing a 9 × 9 feature map. Thus, although the operation is colloquially referred to as a convolution, mathematically, it is a sliding dot product or cross-correlation. Each layer has multiple independent neurons that result in multiple feature maps. All feature maps are stacked along the depth dimension and then passed to the next layer as input. For example, as the first layer has 64 neurons, it turns the 11 × 11 × 1 input patch to a 9 × 9 × 64 volume. Multiple convolution layers are chained to encode the input image into a representation in latent space.

The fully connected (FC) neural network layer takes the encoded representation produced by the CNN layer as input, estimates the center of the input Bragg peak and produces the (*x*, *y*) coordinates as output. In a similar manner to the CNN layer, each FC layer has multiple artificial neurons, each of which has the same number of learnable weights as its input plus one learnable bias. The 3D feature map (*e.g.* 5 × 5 × 4) produced by the last CNN layer is reshaped into a 1D vector before feeding it into the first FC layer. The dot product between the neuron weights and input is computed and added to the bias. Thus, *n* neurons in a given FC layer generate an output vector of dimension *n*, which are passed into the ReLU activation function and then serve as the input of the next layer. As one can see, unlike the CNN neurons that receive input from only a restricted subarea of the previous layer for each convolution point, each neuron in an FC layer is connected to all neurons in the previous layer. The output layer in our design has no activation function (or, equivalently, it applies a linear activation).

The whole process that turns an input Bragg peak patch into two floating point numbers (the coordinates of the peak center) is called a feed-forward pass. The mean-squared error is computed between the model output and ground truth (estimated using pseudo-Voigt fitting) as the model loss. It is important to note here that the ground truth computed using pseudo-Voigt fitting is at best an estimate with some inherent error and the goal of *BraggNN* is to achieve (at least) similar performance to pseudo-Voigt fitting. Training then proceeds as follows. We compute the gradient of the weights of each neuron with respect to the loss function using the chain rule [implemented via automatic differentiation in DL frameworks such as *PyTorch* (Paszke *et al.*, 2019 ▸)]. This process of computing the gradient of learnable weights is called back propagation. The neuron weights are then updated using the gradient descent optimization algorithm (Kingma & Ba, 2014 ▸). Training iterates the feed-forward and back-propagation process on different (Bragg peak patch, ground truth center) pairs many times, until the model no longer makes noticeable progress in minimizing the 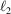 -norm.

We train the *BraggNN* model with a collection of input–output pairs, each with a peak patch as input and the peak center position obtained from pseudo-Voigt fitting as output. Once the *BraggNN* model is trained, we can then apply it to patches obtained from new diffraction data as follows: (1) we use the connected-component labeling algorithm (Fiorio & Gustedt, 1996 ▸; Wu *et al.*, 2005 ▸) to detect connected regions (*i.e.* peaks) in binary digital images. If the region has multiple maxima, indicating the presence of overlapping peaks, the region is discarded. Overlapping peaks will be investigated in a later study. (2) For each region detected in the previous step, we determine the coordinate (row and column index of the image matrix) of its peak (maxima) and crop a patch with a pre-defined size (an odd number, must be the same as that used for training *BraggNN*) with the peak coordinate as the geometric center. Application of the trained *BraggNN* model to such a patch then yields an estimate of the peak position. Given this COM, we then map the position of the peak in the patch back to the diffraction frame based on the location of the patch in the diffraction frame. Each diffraction frame is processed independently, focusing only on 2D shapes of the peaks. In cases of heavily deformed materials, where orientation changes within grains cause the diffraction signal to be present in multiple frames, 3D peak processing would be required and will be investigated in a future study.

### 2.2. Data augmentation

The performance of a deep neural network depends heavily on the quantity, quality and diversity of the data used to train the model. If data are not sufficiently diverse, it is easy to experience overfitting, whereby a network learns a function with very high variance that models the training data perfectly but performs badly on other data. Many application domains, including ours, lack access to large (in terms of both quantity and diversity) and high-quality (accurately annotated) labeled data. Data augmentation is a strategy that enables practitioners to significantly increase the diversity of data available to train their DL models, without actually collecting new data. Data augmentation techniques such as cropping, padding and horizontal flipping are commonly used to train large neural networks for image classification (Cubuk *et al.*, 2018 ▸; Shorten & Khoshgoftaar, 2019 ▸) such as *CIFAR-10* (Krizhevsky, 2009 ▸) and *ImageNet* (Deng *et al.*, 2009 ▸).

Although some existing data-augmentation techniques may be useful in the Bragg peak context to avoid over-fitting, none are useful for training a more generic model (*e.g.* one that generalizes to data outside the training set, or that handles unseen peaks cropped from noisy diffraction frames) because the augmented samples used in the above-mentioned techniques will not be found in practice.

Thus, we introduce a novel physics-inspired data augmentation method that can both avoid overfitting and help to train a more generic model able to deal with imperfect peak cropping from noisy diffraction frames. Specifically, when cropping patches for model training we deviate the peak center from the geometric center randomly by up to ±*m* pixels in the horizontal and ±*n* pixels in the vertical directions.

**Figure 3:**
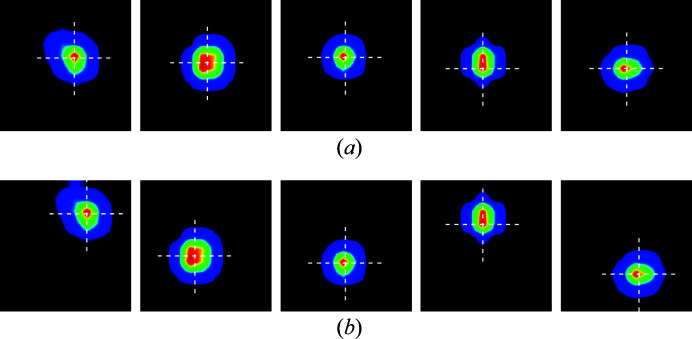
Demonstration of a mini-batch of patches (11 × 11 pixels) with data augmentation for model training. (*a*) The peak maxima is in the center of the patch. (*b*) The peak maxima is intentionally offset by up to ±2 pixels in the horizontal and vertical directions for the same peaks.

Fig. 3 ▸ demonstrates a batch of 10 patches with [Fig. 3 ▸(*a*)] and without [Fig. 3 ▸(*b*)] data augmentation.

This data-augmentation approach helps to train a more general model because, like regularization (Zhang *et al.*, 2016 ▸), it adds prior knowledge (the COM is not always near the geometric center) to a model training and increases training data. It also helps to make the testing dataset statistically more similar to the peak patches that will be encountered during inference in production. The ablation evaluation in Appendix A[App appa] shows the effectiveness of this novel data-augmentation method.

### 2.3. Model training

An important tunable parameter when training a model is the input patch size, as shown in Fig. 2 ▸. The appropriate patch size depends on the pixel size of the detector and size of the diffraction peaks. Best practice is to choose a patch size that can fully cover all valid peaks and still leave 2–4 pixels from peak edge to patch edge for data augmentation. Since the input patch size will determine the size of the neurons in the first fully connected layer, a model trained with one patch size cannot work with another patch size in practice. In the data presented here, 11 × 11 pixel patches were large enough to cover all the different diffraction peaks, but in cases where the diffraction signal has peaks larger than 10 × 10 pixels, *BraggNN* can be retrained with larger patches.

Another tunable parameter is for data augmentation, *i.e.* the interval of displacements (*m* and *n*). We typically choose the same interval size for *m* and *n*. During training, we independently sample (with replacement) a number from the interval for *m* and *n* separately in order to prepare each sample of each mini-batch online.

We implemented our model using the *PyTorch* (Paszke *et al.*, 2019 ▸) ML framework. We train the model for a maximum of 80 000 iterations with a mini-batch size of 512, with validation-based early stopping applied to avoid using an over-fitted model for testing and production use. Training takes about 1 h using one NVIDIA V100 GPU, and less than 1 min using the *Cerebras* (https://www.cerebras.net/) artificial intelligence system (Liu *et al.*, 2021 ▸). In order to (re)train *BraggNN* rapidly using data collected at the early stage of an experiment so as to use it for real-time peak finding from subsequent data near the data source for data reduction and actionable information retrieval using edge computing, we developed (Liu *et al.*, 2021 ▸) an automated workflow to (re)train *BraggNN* using powerful yet remote AI systems in data center or leadership computing facilities.

In the experimental studies reported in this paper we train and evaluate *BraggNN* on a diffraction scan dataset collected using an undeformed bi-crystal gold sample (Shade *et al.*, 2016 ▸) with 1440 frames (0.25° steps over 360°) totaling 69 347 valid peaks. We used 80% of these peaks (55 478) as our training set, 6000 peaks (∼9%) as our validation set for early stopping (Goodfellow *et al.*, 2016 ▸) and the remaining 7869 peaks (∼11%) as a test dataset for model evaluation.

## 3. Results, analysis and discussion

Once the model was trained, we evaluated its performance from two perspectives: (1) we measured the distance (*i.e.* error) between each *BraggNN*-estimated center and the corresponding center obtained via the conventional pseudo-Voigt profile (conventional method); (2) we applied *BraggNN* to an experiment of a different sample, reconstructed grains using peak information by *BraggNN* and compared the reconstructed grain size and position with those reconstructed using conventional methods (Sharma *et al.*, 2012*a* ▸). Note, although we compare *BraggNN* with conventional pseudo-Voigt fitting, the ill-posed inverse problem means that conventional pseudo-Voigt is not the ground truth. Therefore, to evaluate the performance of *BraggNN* in Section 3.2, we compare the results with the reconstructed position of grains in addition to diffraction peak positions. This has two advantages: in case the error in the peak positions is not systematic, the error in the grain positions can be small even if the error in the peak positions is large; and the goal of the analysis is to compute grain positions, so error in grain positions is especially relevant.

### 3.1. Model performance

We start with quantitatively evaluating *BraggNN* by looking at the accuracy of the estimated Bragg positions.

**Figure 4:**
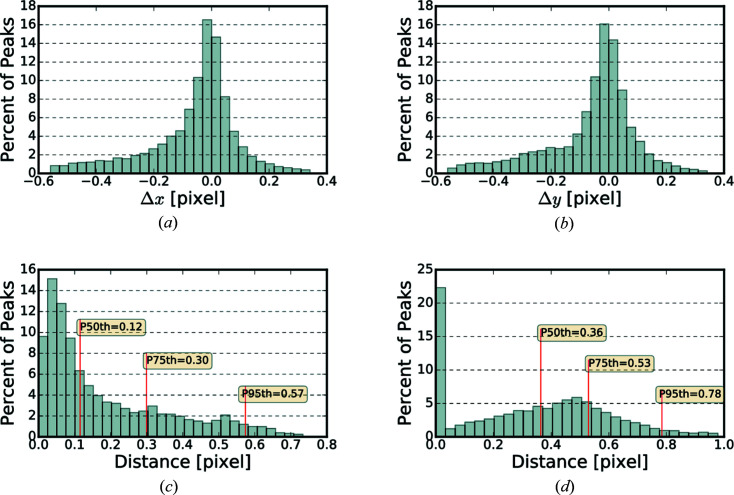
Distribution of difference between peak positions determined using (*a*)–(*c*) *BraggNN* or (*d*) maxima and the conventional pseudo-Voigt fit. (*a*) Difference in horizontal direction, (*b*) difference in vertical direction, (*c*) euclidean distance between peak position and (*d*) euclidean distance between peak positions determined using the maxima position of peaks and conventional pseudo-Voigt fit. *P_n_* in (*c*) and (*d*) denotes the Euclidean distance at the *n*th percentile.

Fig. 4 ▸ shows the distribution of difference between the position of diffraction peaks determined using *BraggNN* [Figs. 4(*a*)–4(*c*)] or peak maxima [Fig. 4(*d*)] and conventional pseudo-Voigt fit.

As quantified using Euclidean distance in Fig. 4 ▸(*c*), most peaks deviate slightly (*e.g.* 75% of peaks deviate less than 0.3 pixels) from the position identified by conventional methods. A computationally advantageous method to guess the COM of peaks is to use just the position of maximum intensity (maxima positions) of the Bragg peaks. However, the differences between maxima positions and conventional method results, shown in Fig. 4 ▸(*d*), are much higher than differences between *BraggNN* and conventional method results in Fig. 4 ▸(*c*), demonstrating the superiority of *BraggNN* over maxima positions.

### 3.2. Reconstruction error analysis

Since the reconstruction of grain positions is our final goal, we also evaluate the trained *BraggNN* on a different dataset (Turner *et al.*, 2016 ▸) and make a comparison with results from the conventional method. The dataset consists of FF- and NF-HEDM data acquired *in situ* during deformation of a Ti–7Al sample. FF-HEDM data were acquired using the same beam configuration at the same location in the sample as NF-HEDM data, thus enabling a one-to-one comparison of the COM position of grains. FF-HEDM directly outputs the COM position of grains, whereas COM positions were calculated from NF-HEDM reconstructions using the voxelized information. A single 2D slice of the specimen was reconstructed using the *MIDAS* software package (Sharma, 2020 ▸; Sharma *et al.*, 2012*b* ▸,*a* ▸) in three different configurations: FF-HEDM reconstruction using *BraggNN*, FF-HEDM reconstruction using conventional pseudo-Voigt fitting and NF-HEDM reconstruction.

**Figure 5:**
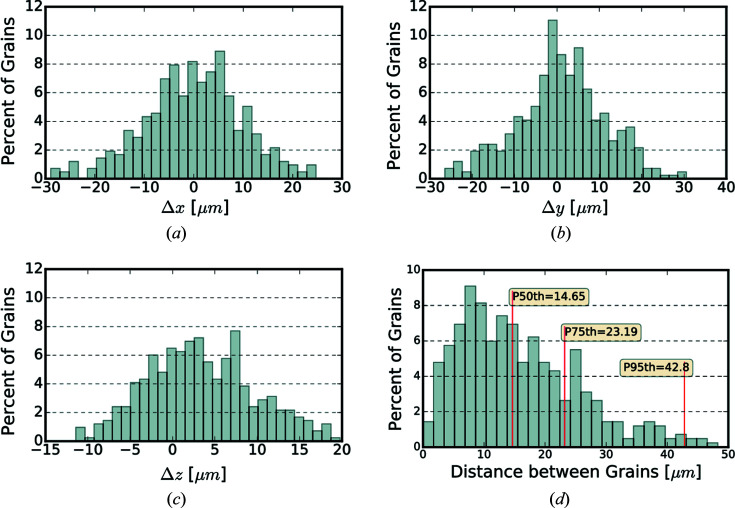
Distribution of position differences between grains when reconstructed using the *BraggNN* peak position and by pseudo-Voigt fitting: (*a*) *x* axis, (*b*) *y* axis, (*c*) *z* axis, (*d*) Euclidean distance. *P_n_* in (*d*) denotes the *n*th percentile.

First we compare the FF-HEDM reconstructions using peak positions obtained from *BraggNN* to the FF-HEDM reconstructions using conventional pseudo-Voigt fitting shown in Fig. 5 ▸. The difference in position in the *x* axis [along the X-ray beam, Fig. 5 ▸(*a*)], *y* axis [horizontal direction perpendicular to the *x* axis, Fig. 5 ▸(*b*)] and *z* axis [vertical direction coincident with the rotation axis, Fig. 5 ▸(*c*)] are centered around 0, implying there is no systematic bias between the two reconstructions. The Euclidean distance between grains reconstructed using *BraggNN* and conventional pseudo-Voigt fitting [Fig. 5 ▸(*d*)] is less than 15 µm for 50% of the grains. This number is similar to the resolution of the FF-HEDM technique (Park *et al.*, 2021 ▸), thus is acceptable. The Euclidean distance is smaller than 50 µm for all the grains.

**Figure 6:**
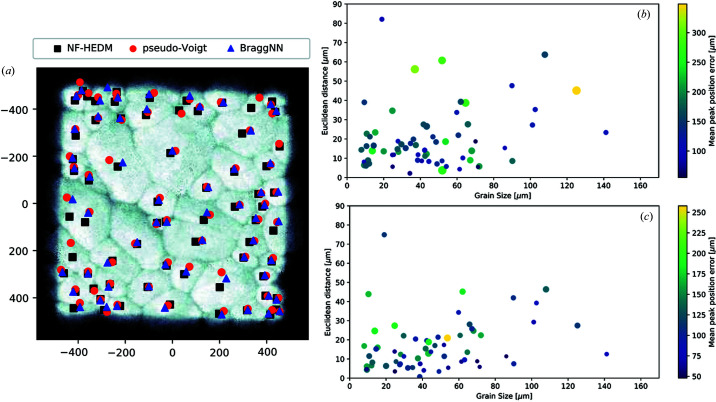
Comparison of *BraggNN*, pseudo-Voigt FF-HEDM and NF-HEDM. (*a*) Grain positions from NF-HEDM (black squares), pseudo-Voigt FF-HEDM (red circles) and *BraggNN* FF-HEDM (blue triangles) overlaid on the NF-HEDM confidence map. (*b*)–(*c*) Difference in the position of grains between pseudo-Voigt FF-HEDM (*b*), *BraggNN* and (*c*) NF-HEDM as a function of grain size. Color of markers in (*b*)–(*c*) represent the mean difference in position of expected and observed diffraction peaks. Size of the markers in (*b*)–(*c*) represent the mean internal angle (see text).

To test the performance of *BraggNN*, we used and compared the grain COMs estimated from NF-HEDM – which results in higher resolution reconstructions by providing a space-filling orientation map – with grain COMs obtained using the FF-HEDM reconstruction technique that is the focus of this paper. A total of 68 grains were identified using NF-HEDM, out of which all the grains could be matched using conventional pseudo-Voigt fitting, but 6 grains were not detected using *BraggNN* because overlapping peaks are ignored. Fig. 6 ▸(*a*) shows the position of grains imaged using the three different reconstruction methods (NF-HEDM, pseudo-Voigt FF-HEDM and *BraggNN* FF-HEDM) overlaid on grain shapes obtained using NF-HEDM. Qualitatively, it can be seen that most of the centroids from *BraggNN* (blue triangles) are situated closer to the NF-HEDM centroids (black squares) than pseudo-Voigt fitting (red circles).

Quantitatively, Fig. 6 ▸(*b*) shows the distance between grains reconstructed using pseudo-Voigt FF-HEDM and NF-HEDM; the mean and median distances are 19.9 and 15.3 µm, respectively. Similarly, Fig. 6 ▸(*c*) shows the distance between grains reconstructed using *BraggNN* and NF-HEDM. The mean and median distances are 17.0 and 13.2 µm. Both mean and median distances are smaller for *BraggNN* by ∼15%, demonstrating a superior performance compared with pseudo-Voigt. Characteristics of grains in FF-HEDM are computed by refining the COM position, crystallographic orientation and strain in each grain using hundreds of diffraction peaks belonging to each grain. This involves computing the expected positions of diffraction peaks using the three grain characteristics and comparing with the observed positions of diffraction peaks. To judge the quality of reconstruction, the mean difference in position of expected and observed diffraction peaks can be used: higher values indicate worse results. The markers in Figs. 6 ▸(*b*) and 6(*c*) are colored according to the mean difference in position of the expected and observed diffraction peaks (on the detector) for each grain for pseudo-Voigt and *BraggNN*, respectively. The average difference for *BraggNN* (116.7 µm) is 28.6% lower than pseudo-Voigt fitting (150.1 µm). The internal angle, another measure of quality of reconstructions, is the mean of the angle between the expected and observed diffraction peaks (in 3D) for each grain. The size of the markers in Figs. 6 ▸(*b*) and 6(*c*) is directly proportional to internal angle of the respective grain with an average internal angle for *BraggNN* (0.083°) 13% better than the pseudo-Voigt fitting (0.094°).

### 3.3. Computational efficiency

Comparison of the reconstructed grain characteristics obtained with *BraggNN* with those obtained with conventional methods reveals similar performances. However, *BraggNN* is much faster. Our highly optimized implementation of 2D pseudo-Voigt fitting, coded in the C programming language, takes about 400 core-seconds to process a dataset of 800 000 peaks on an 2.6 GHz, four-core, Intel Xeon server processor. On the same platform, *BraggNN* takes less than 7 core-seconds to process the dataset, a speedup of 57×. As it is an out-of-the-box solution to run *BraggNN* on a GPU with any DL framework (*i.e.* no extra effort needed to program *BraggNN* for GPU), we also evaluate *BraggNN* on an NVIDIA V100 GPU.

Analysis of the dataset takes only 280 ms, for a speedup of more than 350× relative to the pseudo-Voigt fitting code on a quad-core server CPU (to the best of our knowledge, there is no GPU-accelerated 2D pseudo-Voigt fitting implementation available so far). If no server-class GPU is available near the experiment facility, *BraggNN* on a desktop with an affordable gaming NVIDIA RTX 2080 Ti card only takes about 400 ms, a speedup of 250× relative to running conventional pseudo-Voigt fitting on a high-end workstation CPU. We note that the dataset we used for our evaluation is small, having been collected at only every 0.25° (1440 images for 360°). If we collect with step size of 0.01° (36 000 images for 360°) to assure better angular resolution in peak coordinates, the conventional method will take hundreds of hours to process all peaks whereas *BraggNN* can do it within an hour.

## 4. Future work

*BraggNN* is currently trained to be used for diffraction data consisting of isolated diffraction peaks. Three features of diffraction peaks necessitate the development and extension of *BraggNN* for application to more complex materials:

*Overlapping peaks*. The current implementation of *BraggNN* discards any patches with overlapping peaks (defined currently as patches with more than one maxima). We are extending and training *BraggNN* on larger patches with overlapping peaks to deal with such situations.

*3D peaks from deformed grains*. For heavily deformed grains, the present technique of determining the 2D peak position and computing a weighted COM in 3D can introduce a large error and thus we plan to extend *BraggNN* to carry out the peak localization in 3D.

*Asymmetric peaks*. In extreme cases, the diffraction peaks can no longer be approximated by a pseudo-Voigt shape. We are investigating using multi-modal techniques to determine the peak position with high accuracy and training *BraggNN* to work with such data.

Furthermore, we plan to extend *BraggNN* and apply a DL-based object localization technique directly to diffraction frames to avoid labeling the connection component.

## 5. Conclusions

We have described *BraggNN*, the first ML-based method for precisely characterizing Bragg diffraction peaks in HEDM images. When compared with conventional 2D pseudo-Voigt fitting and using higher resolution NF-HEDM as ground-truth, *BraggNN*-localized peak-based reconstruction can out-perform pseudo-Voigt fitting while running more than 50× faster on a CPU and up to 350× faster on a GPU. The speedup is important for high-resolution, high-throughput and latency-sensitive applications, including real-time analysis and experiment steering (*e.g.* searching the area of interest for multi-scale images).

## Figures and Tables

**Figure 7:**
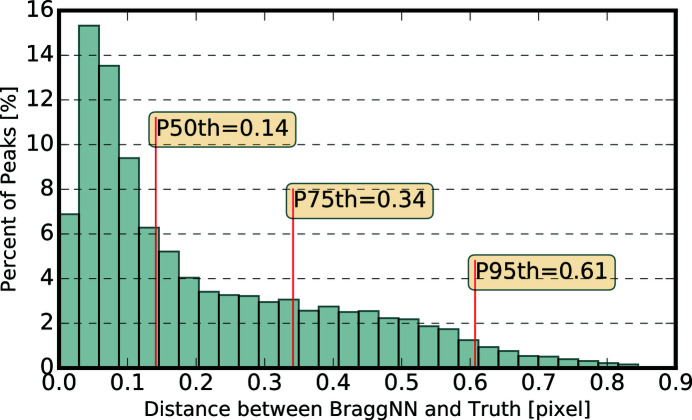
Distribution of difference between peak positions located by *BraggNN* without the non-local self-attention block and conventional pseudo-Voigt fitting.

**Figure 8:**
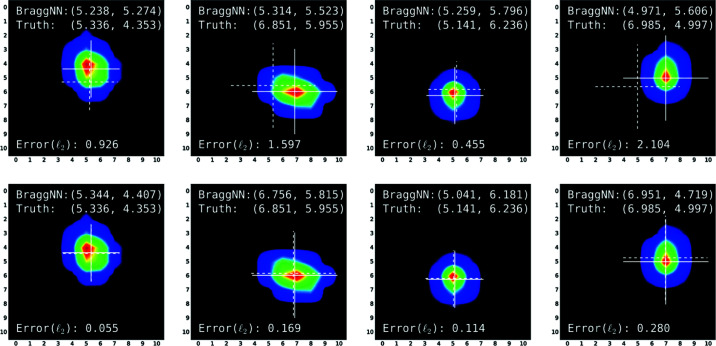
Peaks located by *BraggNN* when peaks deviate from the geometric center. The error is the Euclidean distance ( 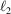 ) between truth (solid-line cross) and *BraggNN* prediction (dotted-line cross). Upper row: using *BraggNN* trained without data augmentation. Bottom row: using *BraggNN* trained with data augmentation for the same peaks as the upper row.

**Figure 9:**
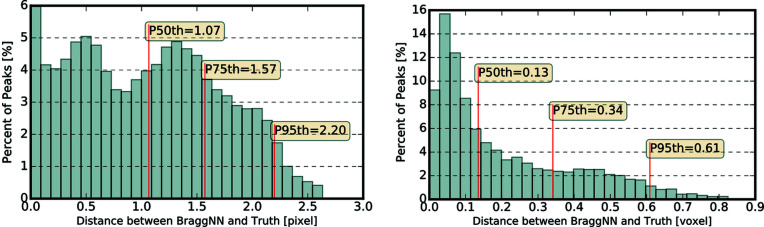
Distribution of difference between peak positions located by *BraggNN* [right: with ( 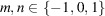 ) or left: without data augmentation] and conventional pseudo-Voigt fitting. *P*_*n*th_ denotes the *n*th percentile.

## Appendix A Ablation study

Here we describe the experiments we used to study the effectiveness of the data augmentation method described in Section 2.2 and the non-local self-attention block in our *BraggNN* architecture design.

### A1. Non-local attention

We used a non-local self-attention block on the feature maps of the first CNN layer for *BraggNN* in order to capture global information on the input patch of peak. The intuition behind this is that a global view at the early layer can help CNN layers better extract feature representation in the latent space for fully connected layers to better approximate its center (Wang *et al.*, 2018 ▸). Here, we conducted an ablation study to show its effectiveness. We trained two models, one with attention block one without, using the same datasets (*i.e.* attention block is the only difference) and then evaluated their estimation accuracy. Fig. 7 ▸ shows the distribution of errors on peak location. It is clear that both the 50th and the 75th percentile deviations are more than 20% worse than Fig. 4 ▸(*c*), where *BraggNN* has the non-local self-attention block, the 95th percentile is about 15% worse.

### A2. Data augmentation

We presented a novel data augmentation method in Section 2.2 to prevent model over-fitting and to address inaccurate patch cropping using the connect component in the model inference phase. In order to study its effectiveness, we trained *BraggNN* on the same dataset with and without augmentation separately. When trained with augmentation, we use an interval of [−1,1] for both *m* and *n*. Fig. 8 ▸ demonstrates three arbitrarily selected cases in our test dataset where the computed peak location deviated from the corresponding patch’s geometric center [*i.e.* (5, 5) for an 11 × 11 pixel patch] in different directions. We can see from the demonstration that *BraggNN* trained with our data augmentation method is able to locate the peak values precisely even when the peak deviates from the geometric center.

In order to quantitatively evaluate the effectiveness of data augmentation, we sample *m* and *n* independently from {−1, 0, 1} when preparing our test dataset to mimic imperfect patch cropping. That is, only 1/3 × 1/3 = 1/9 of the patches have maxima at the geometric center.

Fig. 9 ▸ compares the prediction error on the test dataset in a statistical way. Comparing Fig. 9 ▸(*a*) with Fig. 9 ▸(*b*), we see clear improvement when augmentation is applied for model training. The 50th, 75th and 95th percentile errors are all reduced to about 20% of those obtained when *BraggNN* is trained without data augmentation: a 5× improvement.
